# Myeloid derived suppressor cells contribute to the malignant progression of oral squamous cell carcinoma

**DOI:** 10.1371/journal.pone.0229089

**Published:** 2020-02-24

**Authors:** Xin Pang, Hua-yang Fan, Ya-ling Tang, Sha-sha Wang, Ming-xin Cao, Hao-fan Wang, Lu-ling Dai, Ke Wang, Xiang-hua Yu, Jing-biao Wu, Ya-Jie Tang, Xin-hua Liang

**Affiliations:** 1 State Key Laboratory of Oral Diseases & National Clinical Research Center for Oral Diseases & Department of Oral and Maxillofacial Surgery, West China Hospital of Stomatology, Sichuan University, Chengdu, Sichuan, China; 2 State Key Laboratory of Microbial Technology, Shandong University, Qingdao, China; Chung Shan Medical University, TAIWAN

## Abstract

**Purpose:**

The tumor-related myeloid derived suppressor cells (MDSCs), important immunosuppressive cells in tumor microenvironment, play an important role in the cancer progression. This study is aimed to investigate the crosstalk between MDSCs and oral squamous cell carcinoma (OSCC) cells and their role in the malignant progression of OSCC.

**Methods:**

Immunochemistry (IHC) was used to investigate the expression of CD33 in 200 OSCC, 36 premalignant. CD33^+^ MDSCs were sorted and enriched via magnetic-activated cell sorting (MACS) from OSCC patients or health donor, and their phenotypes were identified by flow cytometry. With a co-culture system of MDSCs and OSCC, the effects of MDSCs on OSCC proliferation, apoptosis, migration invasion, epithelial-mesenchymal transition (EMT), and vasculogenic mimicry formation (VM) formation were assessed, respectively. Besides, peripheral blood mononuclear cells (PBMCs) from health donor were cultured with OSCC supernatant, the level of MDSCs and expressions of Arginase (Arg-1) and inducible nitric oxide synthase (iNOS) were measured.

**Results:**

The number of MDSCs was increased in tumor tissues of OSCC patients, and was positively related to the T stage, pathological grade, lymph node metastasis and poor prognosis. Tumor-related MDSCs of the co-culture system promoted OSCC progression by contributing to cell proliferation, migration and invasion as well as inducing EMT and VM. In turn, OSCC cells had potential to induce MDSCs differentiation from PBMCs and increase the expression of Arg-1 and iNOS.

**Conclusion:**

These indicated that the crosstalk between MDSCs and tumor cells facilitated the malignant progression of OSCC cells and the immune suppressive properties of MDSCs, which may provide new insights into tumor treatment on targeting tumor-associated immunosuppressive cells.

## Introduction

Oral squamous cell carcinoma (OSCC), whose risk factors include alcohol use, tobacco exposure, continued stimulation (areca chewing, for instance), and virus infection, is the most common malignancy among oral cancers [1–3]. The high invasiveness of tumor cells is responsible for the tendency of recurrence and lymph node metastasis in OSCC [4]. Traditional therapeutic approaches including surgery, chemotherapy and radiotherapy failed to improve its five-year survival rate, which is about 50% or lower [5–7]. Thus, identifying new therapeutic targets to inhibit the malignant progression and improve the overall survival (OS) of OSCC patients is in the Spot-LIGHT of researches.

Immune microenvironment consists of a variety of immune cells which can cooperate with each other to inhibit or in contrast be subverted to promote growth and progression of tumor [8, 9]. Among these inmmune cells, myeloid derived suppressor cells (MDSCs), first identified as natural suppressor cells in 1984, which are a heterogeneous group of immature dendritic cells, granulocytes, macrophages, and bone marrow precursor cells, mainly create an immunosuppressive microenvironment [10]. Although there is no uniform biomarkers, MDSCs are commonly been identified to express CD33 and CD11b, and do not express HLA-DR and Lin in human [11–13]. MDSCs can inhibit immune reaction, mediate immune escape, and reduce the effectiveness of tumor immunotherapy through producing soluble factors [14, 15]. Arginase (Arg) derived by MDSCs consumes arginine and subverts T cell signal transduction [14]. Interleukin-10 (IL-10) and transforming growth factor β (TGF-β) secreting by MDSCs serve as critical immune regulators to inhibit T cell proliferation and debilitate immune responses against tumors [16, 17]. Recent findings support that MDSCs can also promote tumor progression by inducing angiogenesis, epithelial-mesenchymal transition (EMT) [18, 19]. Although several studies have shown that MDSCs levels are positively related to histological differentiation, nodal metastasis, and recurrence of OSCC patients [20], the role and mechanism of MDSCs in the malignant progression of OSCC is still unclear.

At present, more and more studies have proved the notion that the interreaction between cancer cells and immune niche can regulate the progression of OSCC. However, there are few studies focus on the crosstalk between MDSCs and tumor cells in the malignant progression of OSCC [21]. Hence, in this study, we sorted CD33^+^ MDSCs from peripheral blood of OSCC patients or healthy donors to established a co-culture system of MDSCs and OSCC cells and determined the effect of MDSCs on proliferation, apoptosis, migration and invasion of OSCC cells, as well as the expression levels of Arg-1 and inducible nitric oxide synthase (iNOS) mRNAs by MDSCs from normal volunteers before and after cultured with the supernatant of OSCC cells. Our study defined a close link between tumor-related MDSCs and the development of OSCC and may validate novel ideas for tumor treatment by targeting tumor-associated immunosuppressive cells.

## Materials and methods

### Patient specimens and blood collection

For immunohistochemistry (IHC) analysis, 200 OSCC and 36 premalignant tissues were obtained from the Department of Oral Pathology, West China Hospital of Stomatology, Sichuan University, between February 2010 and July 2013. None of the patients underwent any types of preoperative (including chemotherapy, radiotherapy or immunotherapy) before surgery. Simultaneously, the clinicopathologic information of patients was collected from the clinical records and pathology reports, including age, gender, site, grade, clinic stage, lymph node metastasis and recurrence.

All subjects gave their written informed consent for inclusion before they participated in the study. The study was conducted in accordance with the Declaration of Helsinki, and West China Hospital of Stomatology (Sichuan University) Ethics Committee approved the protocol before study (No.WCHSIRB-ST-2012-097).

### IHC

IHC was performed as described previously [22]. Briefly, the sections were incubated with 3% hydrogen peroxide and serum for 20 min and 25 min, respectively, then were incubated overnight at 4 °C with the primary antibody against CD33 (proteintech, China) at a dilution of 1:100. Following incubated secondary antibody, all sections were stained with DAB and counterstained with hematoxylin. Then the immunostaining was evaluated with ImageJ2x 2.1.4.7.

### MDSCs isolation and flow cytometry

Peripheral blood mononuclear cells (PBMCs) from blood samples were separated by Ficoll-Hypaque density gradient centrifugation. And MDSCs were sorted from PBMCs by using CD33 labeled magnetically selection monoclonal antibodies (Miltenyi Biotech, Germany). PE mouse anti-human CD33, PE-Cy7 mouse anti-human CD11b, APC mouse anti-human HLA-DR and FITC mouse anti-human LIN (BD Biosciences, USA) were applied to examine the proportion of MDSCs by Flow Cytometry (Cytomic FC500, Beckman). WinMDI was used to analyze samples.

### Cell culture and treatment

Cal-27 and SCC-25 were obtained from State Key Laboratory of Oral Diseases, West China Hospital of Stomatology (Sichuan University) and maintained in DMEM medium (HyClone, USA) with 10% FBS at 37°C with 5% CO_2_. Purified MDSCs from OSCC patients or healthy donors were used for co-culturing with Cal-27 or SCC-25 for the following assays and healthy donors PBMCs were cultured in complete RPMI 1640 medium with 10% FBS or Cal-27/SCC-25-cell culture medium.

### CCK-8 assay

The proliferation of Cal-27 or SCC-25 co-cultured with MDSCs was evaluated by CCK-8 assays. Cal-27/SCC-25 cells were seeded in a 96-well plate at a density of 1×10^3^ cell per well and MDSCs were added at the ratio of 5:1, 1:1 and 1:5, respectively. The co-culture systems were incubated at 37°C with 5% CO_2_. After 6h, 24h, 48h and 72h, CCK-8 kit (Dojindo, Japan) was used to measure the cell viability according to its protocol, and absorbance was measured at 450nm.

### Apoptosis assay

Cal-27 or SCC-25 cells which co-cultured with MDSCs were collected and then 300μL binding buffer and 5μL Annexin V-FITC were added (BOSTER, China). After mixed and stained for 15 min in the dark, 5μL PI (BOSTER, China) was applied. Cells were analyzed using Flow Cytometer.

### Would healing assay

The OSCC cells were co-cultured with MDSCs for 24h, the wound across the diameter of each plate was introduced and then cell migration was observed at 0h, 24h by microscopy. The mean of migration was calculated.

### Transwell invasion assay

Cal-27 or SCC-25 cells were seeded in the upper chamber of Transwell chamber (Millipore, USA) and BD Matrigel in DMEM serum-free medium was coated. Then 100uL cell suspension of MDSCs at concentrations of 1×10^5^/ml derived from OSCC patients or healthy donors was seeded and control group was given. The lower chambers were filled with 500μL medium containing 20% FBS. Cells were incubated at 37°C for 48h. After that, the Transwell chambers were washed, fixed with 5% glutaraldehyde and stained with crystal violet staining solution. Images were obtained with an inverted microscope (Olympus, Japan).

### Real-time PCR

Real-time PCR was performed by TransZol Up Plus RNA Kit per manufacturer’s instructions. Specific primers for Arg-1, iNOS, β-actin, Twist1, Snail, Slug were as follows: Arg-1 forward: 5’-CGCCAAGTCCAGAACCATAG-3’, reverse: 5’- TCCCCATAATCCTTCACATCAC-3’; iNOS forward: 5’- GTGCCTCTATCTTAGCAGCC-3’, reverse: 5’- AGTCCCCTCATCAAAGGTGG-3’; β-actin forward: 5’- AAACACCCCAGCCATGTACGT-3’, reverse: 5’- GTGGTGGTGAAGCTGTAGC-3’; Twist1 forward: 5’- TGTCCGCGTCCCACTAGC-3’, reverse: 5’- TGTCCATTTTCTCCTTCTCTGG -3’; Snail forward: 5’- GACTACCGCTGCTCCATTCCA -3’, reverse: 5’- TCCTCTTCATCACTAATGGGGCTTT -3’; Slug forward: 5’- AGATGCATATTCGGACCCAC -3’, reverse: 5’- CCTCATGTTTGTGCAGGAGA -3’. And 2−ΔΔCt method was applied for relative quantification.

### Vasculogenic mimicry (VM) formation assay

Briefly, 24-well plates were coated with 500uL of Matrigel per well, which was allowed to gel at 37°C for 1 h. OSCC cells and MDSCs were seeded on the Matrigel, and incubated in 5% CO_2_ at 37°C for 48h. Capillary‐like structure formation was captured by an inverted microscope (Olympus, Japan).

### Statistical analyses

All statistical analyses were performed by SPSS 13.0 (SPSS Inc., Chicago, IL, USA) Statistical analysis was performed via two-tailed Student’s t-test or one-way ANOVA. A *P*-value < 0.05 was considered statistically significant.

## Results

### MDSCs infiltration in OSCC tissues was associated with unfavorable prognosis of OSCC patients

**Fig 1:**
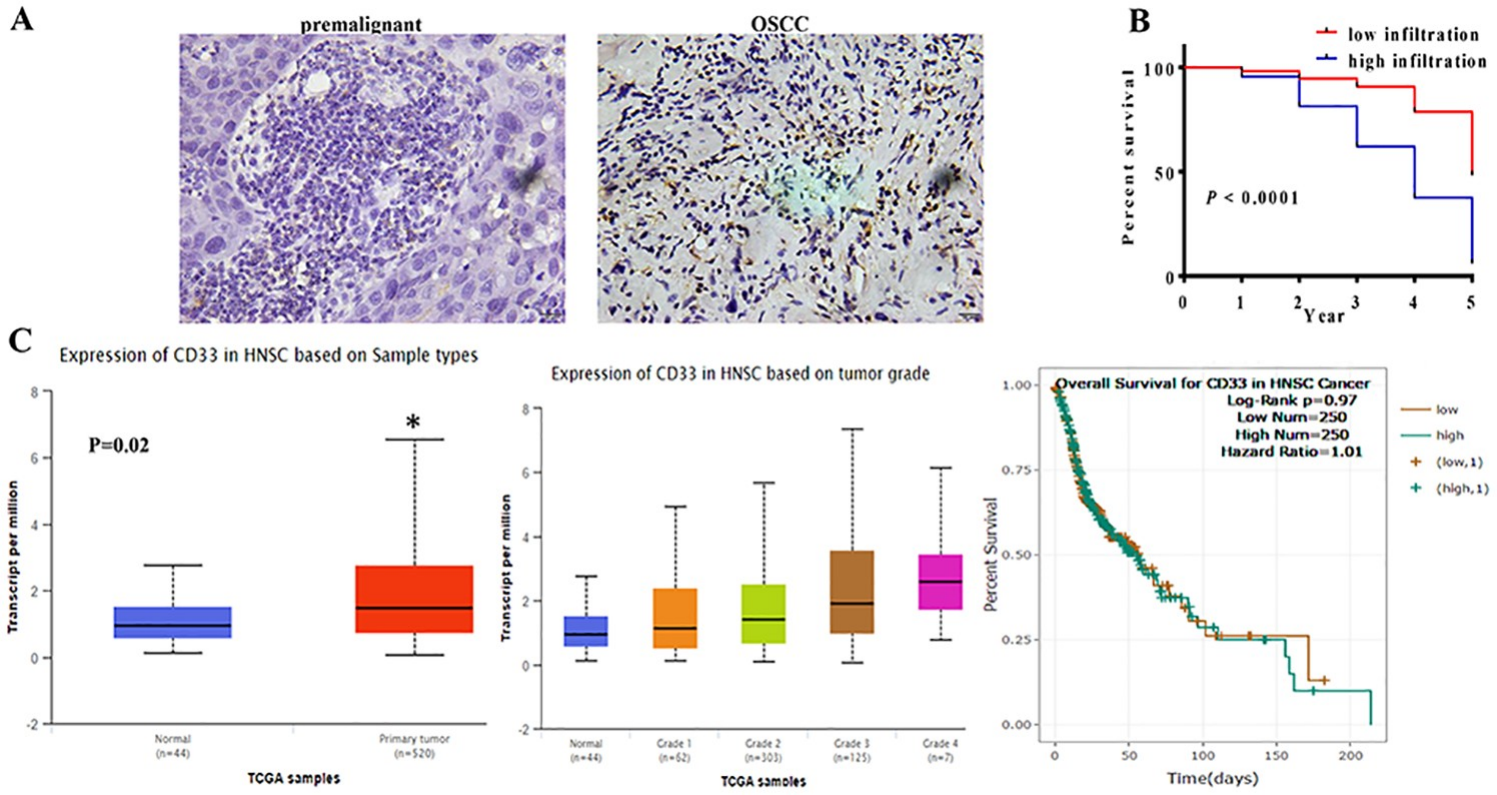
MDSCs in OSCC tissues were associated with the poor prognosis of OSCC patients. **A**. Immunohistochemical staining was use to examine the infiltration of CD33^+^ MDSCs. The data showed that the infiltration of CD33^+^ cells in OSCC tissues was significant higher than in premalignant lesion. Representative figures were shown. **B**. The overall survival curves in OSCC tissue with low or high infiltration of CD33^+^ cells. Survival curves showed that high infiltration of CD33^+^ cells patients showed a lower survival rate than with low infiltration. *P*<0.0001. **C**. The data about CD33 expression in HNSCC patients and the overall survival from database.

To determine the role of MDSCs in OSCC, a total of 200 OSCC patients (143 men and 57 women; mean age, 58 [19–84] years) and 36 premalignant lesions (27 men and 9 women; mean age, 54 [14–65] years) were collected to perform IHC to analysis the expression of CD33 (a MDSCs marker). And no statistically significant difference was noted between two groups in demographic feature. We found that CD33^+^ cells were heterogeneously distributed and preferentially localized at the tumor stroma rather than the epithelium. The number of CD33^+^ cells in OSCC tissues was significantly higher than that in premalignant lesions (Fig 1A). The infiltration of CD33^+^ cells was positively associated with T stage, pathological grade, lymph node metastasis and poor prognosis. However, the MDSCs level was not related with the age, sex, tumor site of patients (*p* > 0.05).

In 200 OSCC, Patients with high infiltration of CD33^+^ cells showed significantly shorter OS (*P* < 0.0001; Fig 1B) than those with low infiltration. And in UALCAN and CHIPBase databases, the expression of CD33 was also up-regulated in HNSCC cases compared with the normal mucous, and the expression of CD33 was associated with the pathological grade and the prognosis of cases, which were similar to our present data (Fig 1C). These observations implicated the unfavorable role of MDSCs infiltration in the prognosis for OSCC patients.

### Tumor-associated MDSCs confer proliferative potential on OSCC cells

**Fig 2:**
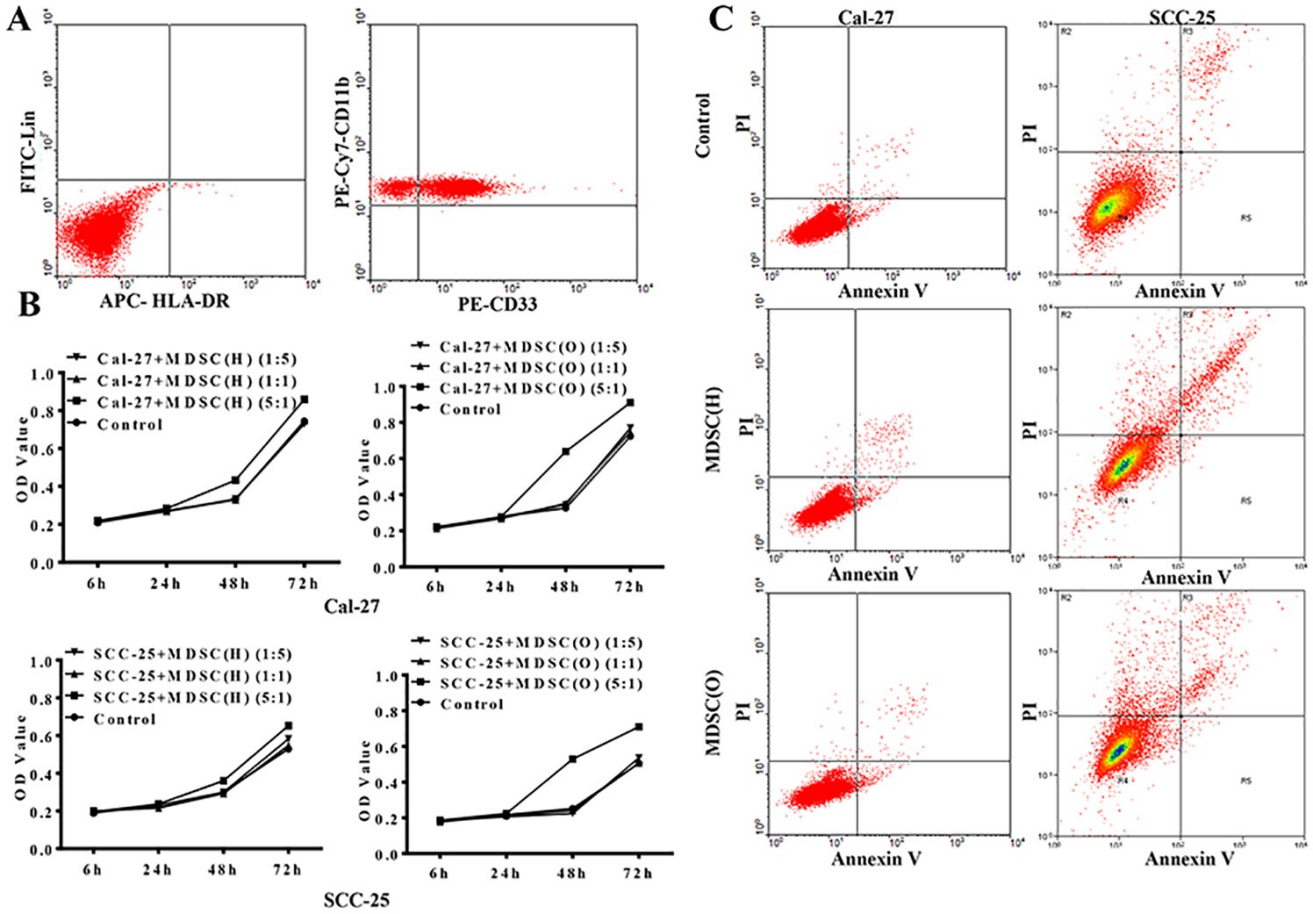
Tumor-associated MDSCs confer proliferative potential on OSCC cells. **A**. CD11b, CD33, Lin, HLA-DR were used to identify the purity of MDSCs sorted from PBMCs with multiparametric flow cytometry analysis. **B**. CCK8 assay was used to examine the cell growth rates cells in control, MDSCs co-culture group, respectively. The data showed that the cell growth rates of OSCC cells were significantly increased when co-cultured with MDSC from OSCC patients, compared with the control and co-cultured with MDSCs from health donors. Error bars represent the mean ± SD of triplicate experiments. * P <0.05. **C**. Flow cytometry showed cell apoptosis in control and MDSCs co-culture group of Cal-27 and SCC-25. The data showed that there was no difference among three groups. Representative figures of three independent experiments were shown.

To assess whether tumor-associated MDSCs affected proliferation and apoptosis of OSCC cells, CD33 magnetic activated cell sorting (MACS) was firstly used to isolate MDSCs from peripheral blood of OSCC patients and healthy donors, respectively. And 84% purity of sorted CD33^+^ CD11b^+^ Lin^-^ HLA-DR^-^ cells was obtained (Fig 2A).

Then, MDSCs sorted from OSCC patients [MDSCs (O)] or healthy donors [MDSCs (H)] were co-cultured with Cal-27 or SCC-25 cells for 24 to 72 hours, respectively. CCK8 assays showed that Cal-27 and SCC-25 cells proliferation was increased after co-cultured with MDSCs at the ratio of 5:1 for 48 or 72 hours (P<0.05). And the proliferation of OSCC cells was strongly enhanced after co-cultured with OSCC-derived MDSCs but slightly increased when treated with MDSCs from healthy donors (Fig 2B). However, the apoptosis of Cal-27 and SCC-25 cells did not differ significantly after co-cultured with MDSCs from OSCC patients or healthy donors (Fig 2C). Therefore, our results showed tumor derived MDSCs could enhance the proliferation of OSCC cells but had no effect on their apoptosis.

### Tumor-associated MDSCs promoted migration and invasion on OSCC cells

**Fig 3:**
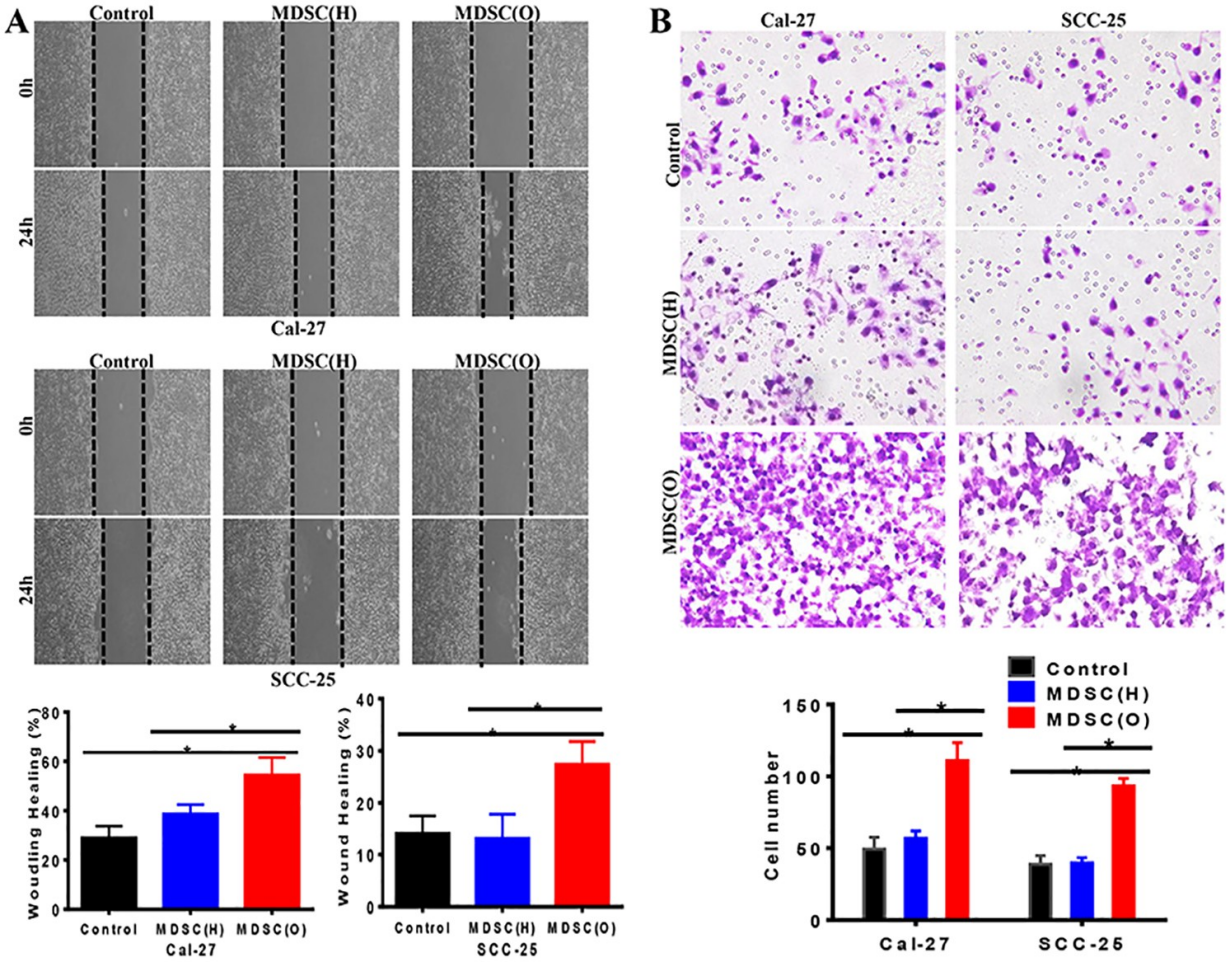
Tumor-associated MDSCs promoted migration and invasion on OSCC cells. **A**. Migration assay examined the cell migration ability in control and MDSCs co-culture group of Cal-27 and SCC-25, respectively. Representative figures were shown. The migration ability of OSCC cells co-cultured with OSCC MDSCs was significantly enhanced compared with the control or OSCC cells co-cultured with MDSCs from health donors. The mean was derived from cell counts of 3 fields, and each experiment was repeated 3 times. Error bars represent the mean ± SD of triplicate experiments. * P <0.05. **B**. Invasion assay examined the cell invasion ability in control and MDSCs co-culture group of Cal-27 and SCC-25, respectively. Representative figures were shown. The invasion ability of OSCC cells co-cultured with OSCC MDSCs was significantly increased compared with control or OSCC cells co-cultured with MDSCs from health donors. The mean was derived from cell counts of 3 fields, and each experiment was repeated 3 times. Error bars represent the mean ± SD of triplicate experiments. *P<0.05.

With the co-culture system, we next evaluated the role of MDSCs in the migratory ability of Cal-27 and SCC-25 cells by wound healing assay. The data showed that there was a significant increase of the migration of Cal-27 and SCC-25 cells after co-cultured with tumor-associated MDSCs for 24 hours compared with MDSCs from healthy donors (P<0.05) (Fig 3A). Further, Transwell invasion assay showed that the invasion of OSCC cells was significantly enhanced in OSCC-MDSCs group compared with healthy donor-MDSCs group (*P*<0.05) (Fig 3B). Therefore, our results demonstrated an obvious enhancement migration and invasion of OSCC cells induced by tumor derived MDSCs.

### Tumor-associated MDSCs induced EMT of OSCC cells

**Fig 4:**
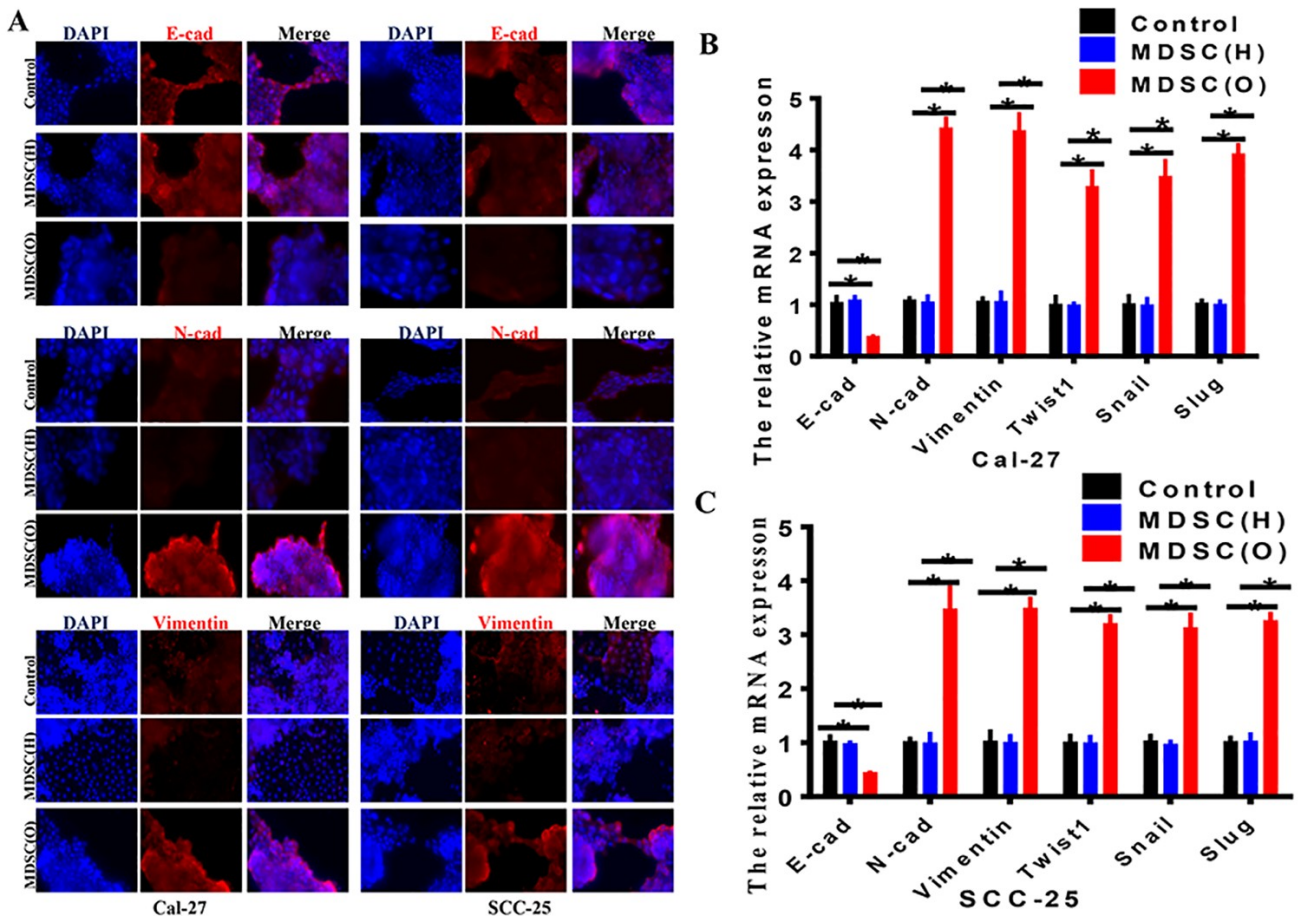
Tumor-associated MDSCs induced EMT of OSCC cells. **A**. Immunofluorescence staining showed the protein expression of E-cadherin, N-cadherin and Vimentin in control and co-culture group of Cal-27 and SCC-25. The data showed the protein levels of N-cadherin and Vimentn were up-regulated in OSCC cells co-cultured with OSCC derived MDSCs. The change of E-cadherin expression was reverse. Representative figures were shown. **B**. RT-PCR showed the mRNA expression of E-cadherin, N-cadherin, Vimentin, Twist1, Snail and Slug in control or co-culture group of Cal-27 cells. The data showed that the mRNA expression of N-cadherin, Vimentin, Twist1, Snail and Slug was enhanced in cells co-cultured with OSCC derived MDSCs. The change of E-cadherin expression was reverse. Each experiment was repeated 3 times. Error bars represent the mean ± SD of triplicate experiments. * *P* <0.05. **C**. RT-PCR showed the mRNA expression of E-cadherin, N-cadherin, Vimentin, Twist1, Snail, Slug in control or co-culture group of SCC-25 cells. The data showed that the mRNA expression of N-cadherin, Vimentin, Twist1, Snail and Slug increased in cells co-cultured with OSCC derived MDSCs. The change of E-cadherin expression was reverse. Each experiment was repeated 3 times. Error bars represent the mean ± SD of triplicate experiments. * *P* <0.05.

To investigate the role of MDSCs in inducing EMT of OSCC cells, we examined both epithelial and mesenchymal markers by immunofluorescence staining (Fig 4A) and RT-PCR (Fig 4B and 4C) in OSCC cells co-cultured with MDSCs from OSCC patients or healthy donors, respectively. As we can see, compared with OSCC cells with healthy donor MDSCs, OSCC cells with MDSCs from OSCC patients exhibited a significant down-regulation of E-cadherin from cell-cell contacts; meanwhile the mesenchymal markers N-cadherin and Vimentin were dramatically upregulated (Fig 4A–4C). We further examined the expression of other known EMT-associated transcription factors. The data showed that the endogenous mRNA levels of Twist1, Snail, Slug were elevated in response to stimulation of MDSCs from OSCC patients in a variable extent (Fig 4B and 4C). Together, these results indicated that tumor-associated MDSCs may be a novel inducer of EMT in Cal-27 and SCC-25 cells.

### Tumor-associated MDSCs promoted VM formation of OSCC cells

**Fig 5:**
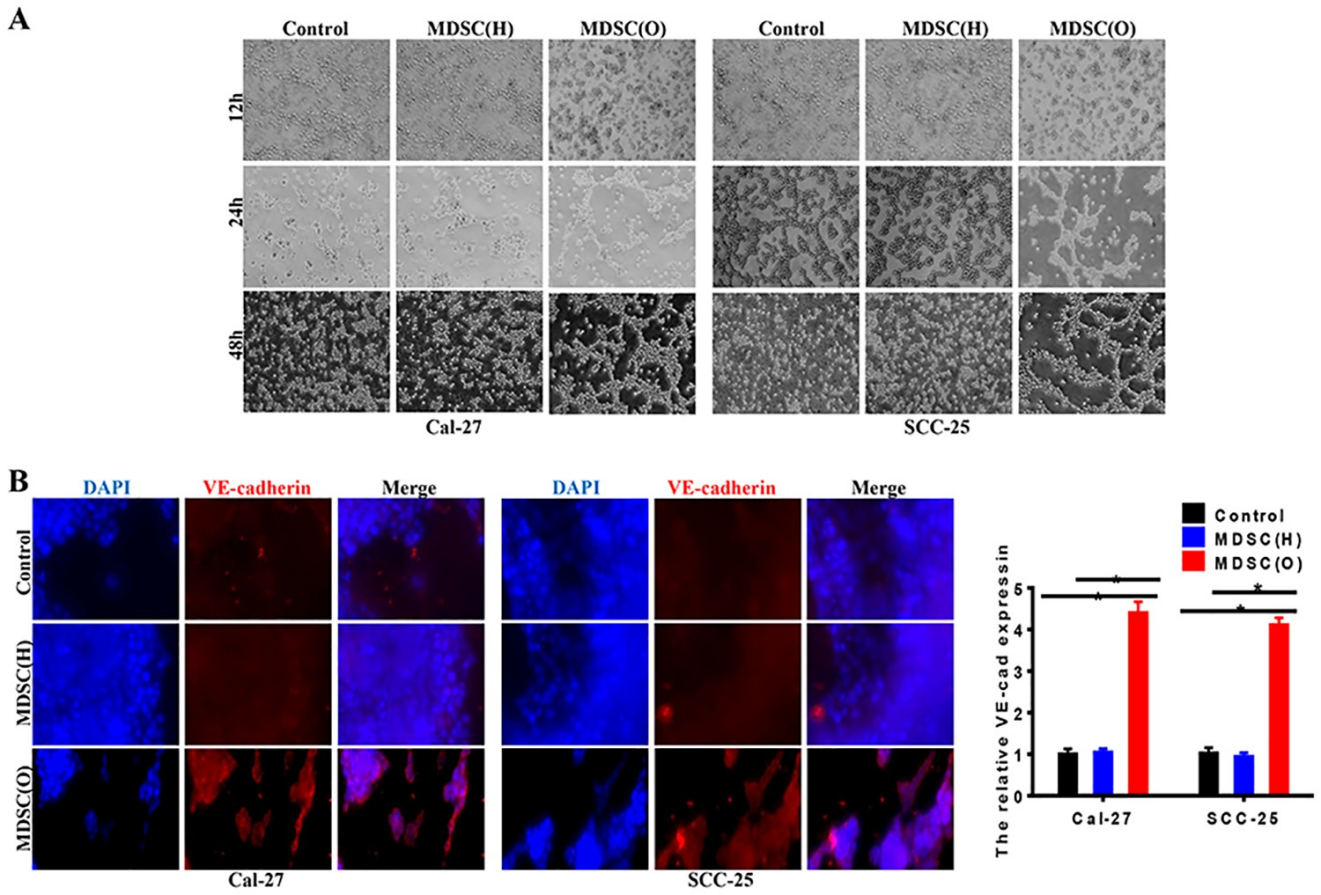
Tumor-associated MDSCs promoted VM of OSCC cells. **A**. Tube-like structure formation on Matrigel in Cal-27 and SCC-25 cells. OSCC cells co-cultured with OSCC derived MDSCs showed a stronger ability of VM formation compared with the control and cells co-cultured with health MDSCs. **B**. Immunofluorescence staining and RT-PCR assessed the effect of MDSCs on VE-cadherin protein and mRNA expression in OSCC cell lines, respectively. The data showed that OSCC derived MDSCs enhanced the level of VE-cadherin in SACC cells in both protein and mRNA levels. Error bars represent the mean ± SD of triplicate experiments. * *P* <0.05.

VM formation in epithelial cancer is assumed to be associated with the EMT process, and the regulators that contribute to EMT may also modulate VM formation [23]. Here, we wonder whether tumor-associated MDSCs could promote VM formation of OSCC cells. We used a well-established in vitro 3-D culture model to investigate VM formation. Results showed that after 48h, OSCC cells treated with tumor-associated MDSCs formed the typical vessel-like structures; whereas MDSCs from healthy-donor could not (Fig 5A). In addition, compared with OSCC cells with healthy-donor MDSCs, tumor-associated MDSCs increased the expression of vascular endothelial (VE)-cadherin of OSCC cells, which were characteristics of endothelial cells (Fig 5B). These indicated that OSCC derived MDSCs might promote VM formation of OSCC cells through induction of EMT.

### OSCC cells induced MDSCs differentiation with immunesuppressive phenotype from PBMCs

**Fig 6:**
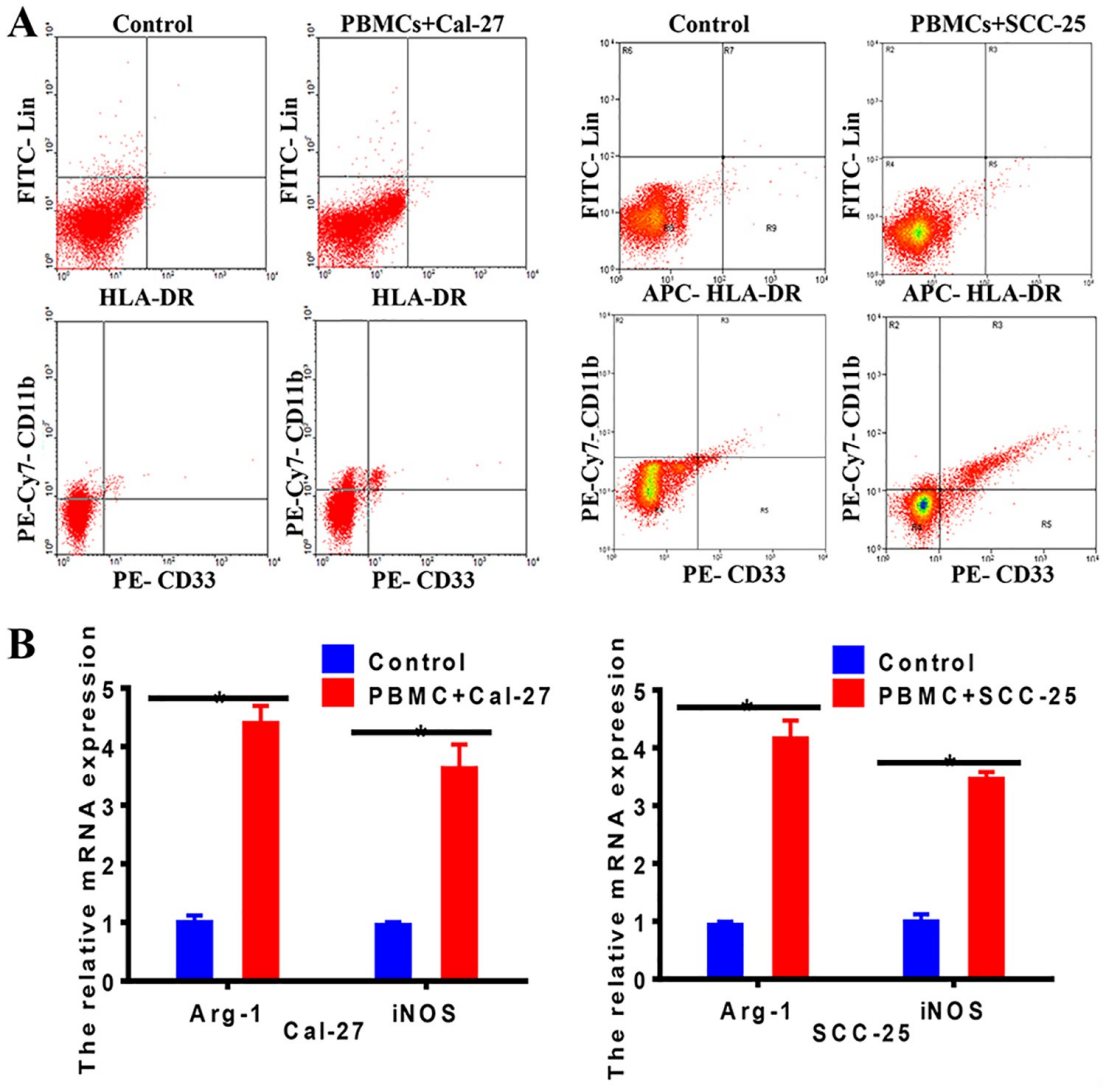
OSCC cells induced MDSCs differentiation with immunesuppressive phenotype from PBMCs. **A**. CD11b, CD33, Lin, HLA-DR were used to identify the purity of MDSCs sorted from PBMCs with multiparametric flow cytometry analysis. The data showed that OSCC increased the rate of MDSCs in PBMCs after co-cultured 24h. Representative figures of three independent experiments were shown. **B**. RT-PCR was applied to examine the mRNA expression of Arg-1 and iNOS in MDSC. The data showed that the mRNA expression of Arg-1 and iNOS increased in MDSC after co-cultured with OSCC. Each experiment was repeated 3 times. Error bars represent the mean ± SD of triplicate experiments. * *P* <0.05.

Studies have manifested that MDSCs can be recruited to tumor microenvironment to accelerate tumor progression [24, 25]. To confirm whether OSCC cells induced PBMCs differentiation into MDSCs and enhanced immunesuppression phenotype of MDSCs, we collected PBMCs from healthy donors and cultured them with OSCC cells culture medium for 24 hours. The proportion of MDSCs was then quantified by flow cytometric analysis. As shown in Fig 6A, a significant expansion of CD33^+^ CD11b^+^ Lin^-^ HLA-DR^-^ MDSCs was observed in PBMCs cultured with OSCC cells culture medium, compared with control group (*P*<0.05). Then, we analyzed whether these OSCC-induced MDSCs obtained immunesuppressive abilities. Strikingly, the mRNA expression of Arg-1 and iNOS was significantly increased in healthy donor MDSCs cultured with Cal-27 or SCC-25 cells as determined by qRT-PCR (Fig 6B). Our data confirmed Cal-27 and SCC-25 cells converted healthy donor-MDSCs to tumor-associated MDSCs with immunesuppressive phenotype.

## Discussion

It is worth noting that immune microenvironment has been implicated in the initiation and progression of cancers. MDSCs facilitate immune evasion of tumor cells, as well as decrease the efficacy of immunotherapy [26]. Here we investigated a crosstalk between tumor-related MDSCs and OSCC cells. The data showed that the increased MDSCs level in tumor was obviously associated with the poor prognosis of OSCC patients. Tumor-associated MDSCs conferred proliferative, migrative and invasive potential of OSCC cells, as well as induced EMT and VM of OSCC cells. In addition, OSCC cells induced MDSCs differentiation with immune suppressive phenotype from PBMCs and enhanced the secretion of Arg-1 and iNOS of MDSCs. These findings provided insights into tumor treatment by reducing tumor-associated immunosuppressive cells.

Early evidence supported that MDSCs level was associated with clinical stage and distant metastasis in many human cancers including HNSCC, and surgical excision of tumors could decrease the concentration of peripheral blood MDSCs [27–30, 29]. Hence, we addressed the number of infiltrated MDSCs by IHC in OSCC tissues and found that MDSCs were increased significantly in tumor stroma that was near to OSCC cells. Consistently, researches showed MDSCs were significantly increased in the stroma of renal cell carcinoma and glioblastoma multiforme as assayed by flow cytometry [31–33]. And Horikawa et al. [34] also identified an increased MDSCs infiltration in ovarian cancer which was inversely correlated with intratumor CD8^+^ T cell numbers and inferior overall survival (OS). Further, zhang et al. [35] conducted a meta-analysis with eight studies contained 442 solid cancer patients, and found MDSCs were associated with poor OS, disease-free survival (DFS) and progression-free survival (PFS). Thereby, we hypothesized that a crosstalk between OSCC cells and MDSCs may accelerate the malignant progression of OSCC.

To address the relationship between OSCC and MDSCs, we firstly analyzed the role of MDSCs in the development of OSCC. Here, our data revealed that OSCC associated MDSCs could facilitate OSCC progression by directly enhancing the proliferation, migration and invasion of OSCC cells, whereas the apoptotic ability of OSCC cells was not affected. Similarly, Toh et al. [36] showed that granulocytic MDSCs stimulated melanomas cells proliferation in a dose-dependent manner in vitro. It has also been demonstrated that MDSCs directly enhanced B16 melanoma cell proliferation by mTOR signaling. Also, MDSCs depletion resulted in a decrease the of cancer cells proliferation in a murine model of melanoma as revealed with IHC. [37]. Zhao et al. [38] found the stimulatory effects of lal^−/−^ Ly6G^+^ MDSCs on B16 melanoma cell migration after co-cultured 24h. In mammary carcinomas, Gr-1^+^ CD11b^+^ MDSCs were recruited to the invasive front of tumor, and the invasion ability of mammary carcinomas cell line, 4T1, displayed a significant increase after co-cultured with MDSCs overnight [39]. These indicated that MDSCs could promote the progression of tumor by directly stimulating OSCC cells proliferation, migration and invasion apart from suppression of immune surveillance.

Interestingly, we also indicated that OSCC associated MDSCs accelerated malignant progression of OSCC by enhancing EMT essential for epithelial tumor metastasis. In accordance with our results, researches have demonstrated that CXCR2^+^ MDSCs were predominately expanded and recruited in breast cancer and could boost EMT of breast cancer cells dependently on IL-6/STAT3 signaling [40]. And the elimination of MDSCs diminished tumor metastasis in breast carcinoma model [41]. Further, studies also demonstrated that MDSCs induced EMT through secreting EGF, TGF-β1, and HGF. Once MDSCs were depleted, the expression of S100A4 and Vimentin which downregulated the expression of E-cadherin and promoted EMT would be decreased in a murine melanoma model [36]. EMT has been proved to contribute to the VM which is described as a process that aggressive tumor cells mimic the endothelial cells to form microvascular tubes and has been reported to promote progression of cancer [42]. Our previous work found that the VEGFA induced VM formation through regulating EMT to fuel the migration and invasion of salivary adenoid cystic carcinoma [43]. Recently, several studies have revealed that immune cells are involved in the VM. Rong et al. [44] showed that tumor-associated macrophages (TAMs) could were capable of driving formation of VM in glioblastoma multiforme via COX-2 dependent manner. Similarly, in glioma, CD163^+^ TAMs induce the VM by enhancing the secretion of IL-6 via PKC pathway [45]. However, at present, there is still lake of study on the role of MDSCs in the VM formation of tumor. In this study, we identified that OSCC derived MDSCs facilitated the VM formation of OSCC cells. Together with our data, we concluded that cancer related MDSCs could promote OSCC malignant progression by inducing EMT and VM formation.

Thus, our findings identified that MDSCs played a critical role in the malignant in the progression of OSCC, subsequently, the effect of OSCC cells on MDSCs has also been explored. We showed that OSCC cells induced MDSCs differentiation from PBMCs of healthy donors. In line with our data, Karakasheva et al. [46] found a significantly increased level of CD38^+^ monocytic MDSCs in PBMCs of colorectal cancer patients compared with healthy donors. Lechner et al. [47] assessed the ability of over 100 human tumor cell lines to induce MDSCs from PBMCs of healthy donor via co-culture, and found CD33^+^ MDSCs be induced by all types of cell lines including cells from head and neck squamous cell carcinomas, but exception of those derived from breast cancer.

The Arg-1 and iNOS were the crucial immunosuppressive mediators in immunesuppression induced by MDSCs. The activity of Arg-1 and iNOS cause the decomposition of arginine, which leads to T cell cycle arrest in G0-G1, and finally leads to T cells anergy [48]. Zhang et al. [49] identified that HLA-DR^-^ CD33^+^ CD11b^+^ MDSCs from NK/T cell lymphoma patients expressed higher levels of Arg-1 and iNOS compared with the levels of MDSCs from healthy donors and strongly inhibited the CD4^+^ T cell proliferation but slightly suppressed CD8^+^ T cell proliferation. Namdar et al. [50] showed that Foxp3 vaccination suppressed MDSCs activity via a significant decrease of Arg-1 and iNOS to reduction of melanoma growth in a murine model. Cao et al. [51] demonstrated that L-Arg supplementation significantly inhibited tumor growth by reduction of MDSCs, and enhanced innate and adaptive immune responses in melanoma mice model. Hence, we tested the expression of iNOS and Arg-1 in PBMCs after co-cultures with OSCC, and found OSCC could enhance the levels of iNOS and Arg-1, providing evidence that tumor cells could educate the immune cells to immunosuppressive phenotype.

## Conclusions

Overall, our study demonstrated that MDSCs, the most potent inhibitors of T cells responses historically, were closely associated with the malignant progression of OSCC by promoting the proliferation, migration, invasion, and EMT as well as VM formation. And in turn, OSCC cells could also promote MDSCs increment and enhance immune suppressive function of MDSCs. These findings hinted that targeting MDSCs might a new manner to therapy OSCC.
