# Correction: Myeloid derived suppressor cells contribute to the malignant progression of oral squamous cell carcinoma

**DOI:** 10.1371/journal.pone.0355520

**Published:** 2026-08-10

**Authors:** Xin Pang, Hua-yang Fan, Ya-ling Tang, Sha-sha Wang, Ming-xin Cao, Hao-fan Wang, Lu-ling Dai, Ke Wang, Xiang-hua Yu, Jing-biao Wu, Ya-Jie Tang, Xin-hua Liang

After this article [1] was published, concerns were raised with Figs 3 and 5. Specifically:

- In Fig 3A, the Cal-27 MDSC(O) 0h panel appears to overlap with the Cal-27 Control 0h and Cal-27 MDSC(H) 0h panels.
- In Fig 5A, the Cal-27 Control 24h and Cal-27 MDSC(H) 24h panels appear to overlap.
- In Fig 5A, the SCC-25 MDSC(O) 48h panel appears to overlaps with the SACC-LM 48h panel in Fig 2A of [2], and these panels appear to represent different cell lines.

Co-first author XP stated that all three of the Cal-27 0h panels in Fig 3A are incorrect. They provided an updated version of Fig 3 with the correct Cal-27 0h panels in Fig 3A from the original experiments reported in [1]. The underlying data for Fig 3 are provided here in S1 and S3 Files.

Co-first author XP stated that the Cal-27 MDSC(H) 24h panel in Fig 5A is incorrect. They provided an updated version of Fig 5 with the correct Cal-27 MDSC(H) 24h panel in Fig 5A from the original experiments reported in [1]. The underlying data for Fig 5 are provided here in S2 File. Co-first author XP also stated that the SCC-25 MDSC(O) 48h panel in Fig 5A of [1] is correct and the SACC-LM 48h panel in Fig 2A of [2] is incorrect.

A member of the *PLOS One* Editorial Board reviewed the updated Figs 3 and 5 and stated that they support the results in [1].

The raw data underlying Figs 1-6 are missing from the list of Supporting Information. The authors have provided the data as Supporting Information S1-S4 Files. With this correction, all relevant data are now provided.

The SCC-25 MDSC(O) 48h panel in Fig 5A in [1] appears similar to content previously published in [2] under a CC BY license. The reused content was modified. The SCC-25 MDSC(O) 48h panel in Fig 5A in [1] is subject to the license that applies to the original article; please provide due attribution to the original publication when referring to this content.

## Supporting information

S1 File Individual-level underlying data and images for Fig 3B, and underlying image for the corrected Fig 3A. (ZIP)

S2 File Individual-level underlying data and images for Fig 5B, and underlying image for the corrected Fig 5A. (ZIP)

S3 File Individual-level underlying quantitative data for Figs 1B-C, 2A, 2B (SCC-25), 2C, 4B-C, 6A-B, and for the corrected Fig 3A. (RAR)

S4 File Individual-level underlying quantitative data for Fig 2B (Cal-27). (RAR)

**Fig 3:**
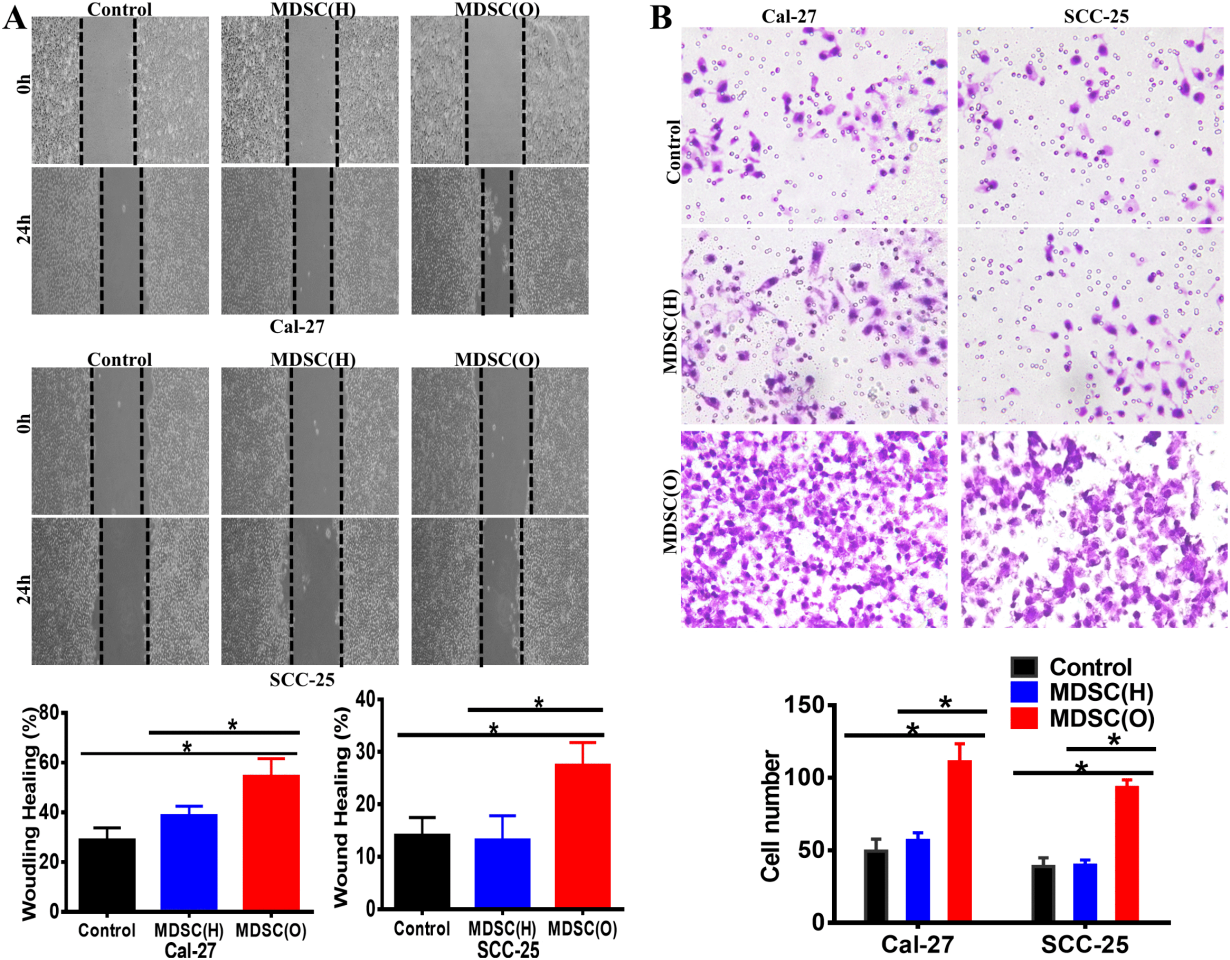
Tumor-associated MDSCs promoted migration and invasion on OSCC cells. **A.** Migration assay examined the cell migration ability in control and MDSCs co-culture group of Cal-27 and SCC-25, respectively. Representative figures were shown. The migration ability of OSCC cells co-cultured with OSCC MDSCs was significantly enhanced compared with the control or OSCC cells co-cultured with MDSCs from health donors. The mean was derived from cell counts of 3 fields, and each experiment was repeated 3 times. Error bars represent the mean ± SD of triplicate experiments. *P < 0.05. **B.** Invasion assay examined the cell invasion ability in control and MDSCs co-culture group of Cal-27 and SCC-25, respectively. Representative figures were shown. The invasion ability of OSCC cells co-cultured with OSCC MDSCs was significantly increased compared with control or OSCC cells co-cultured with MDSCs from health donors. The mean was derived from cell counts of 3 fields, and each experiment was repeated 3 times. Error bars represent the mean ± SD of triplicate experiments. *P < 0.05.

**Fig 5:**
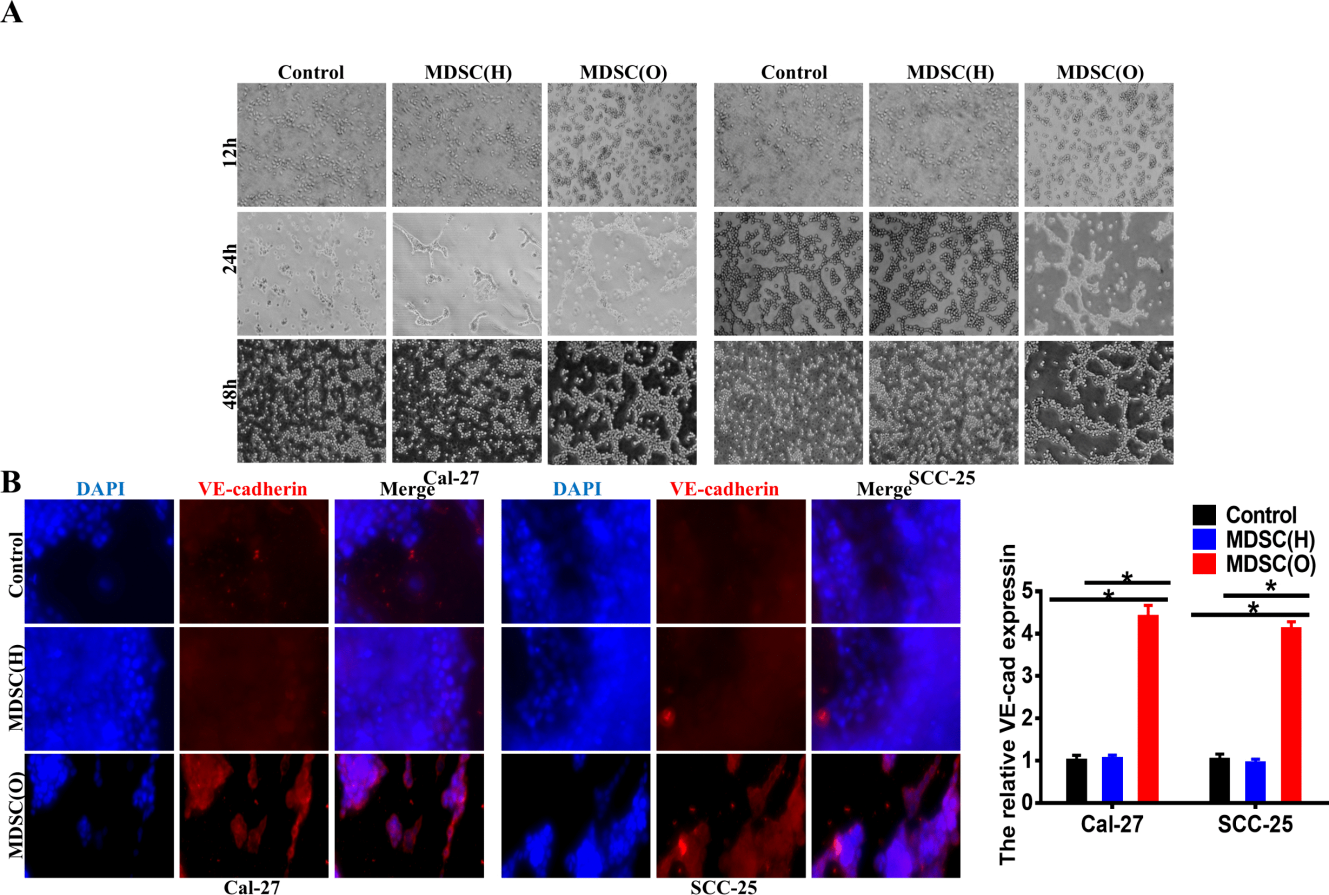
Tumor-associated MDSCs promoted VM of OSCC cells. **A.** Tube-like structure formation on Matrigel in Cal 27 and SCC-25 cells. OSCC cells co-cultured with OSCC derived MDSCs showed a stronger ability of VM formation compared with the control and cells co-cultured with health MDSCs. **B.** Immunofluorescence staining and RT-PCR assessed the effect of MDSCs on VE-cadherin protein and mRNA expression in OSCC cell lines, respectively. The data showed that OSCC derived MDSCs enhanced the level of VE-cadherin in SACC cells in both protein and mRNA levels. Error bars represent the mean ± SD of triplicate experiments. * *P* < 0.05.
